# Supplementary material for: Classifying cell cycle states and a quiescent-like G0 state using single-cell transcriptomics
Source: bioRxiv. 2024 Sep 22:2024.04.16.589816. Originally published 2024 Apr 20. Preprint. [Version 2] doi: 10.1101/2024.04.16.589816 (PMC11042294; doi:10.1101/2024.04.16.589816)
Supplement: Supplementary file 1 [file media-1.docx]

**Supplemental information for Classifying cell cycle states and a quiescent-like G0 state using single-cell transcriptomics**

Samantha A. O’Connor^1^, Leonor Garcia^2^, Anoop P. Patel^3,4^, Benjamin B. Bartelle^1^, Jean-Philippe Hugnot^2^, Patrick J. Paddison^5^, Christopher L. Plaisier^1,^*

^1 ^School of Biological and Health Systems Engineering, Arizona State University, Tempe AZ, USA

^2^ Institut de Génomique Fonctionnelle, Université de Montpellier, CNRS, INSERM, 141 rue de la Cardonille, 34091, Montpellier, France.

^3^ Brotman-Baty Institute for Precision Medicine, University of Washington, Seattle, WA, USA.

^4 ^Department of Neurosurgery, Preston Robert Tisch Brain Tumor Center, Duke University, Durham, NC, USA.

^5^ Human Biology Division, Fred Hutchinson Cancer Research Center, Seattle WA, USA

* To whom correspondence should be addressed: Christopher Plaisier, E-mail:  [plaisier@asu.edu](mailto:plaisier@asu.edu), Phone: (480) 965-6832, Address:  P.O. Box 879709, Tempe, AZ 87287-9709

Table of Contents 

Supplemental Methods	3

**Human neural stem cells**	3

**Glioblastoma tumor and cancer stem cells**	3

**Low grade glioma IDH mutant tumor cells**	3

**Human neural stem cells from fetal tissue at post-conception weeks 3-12**	3

**Human cells from fetal tissue at post-conception weeks 3-12**	4

**Atlas of the developing human telencephalon**	4

**Atlas of adult neurogenesis in the ventricular-subventricular zone (V-SVZ)**	4

**Activated and quiescent neural stem cells**	4

**Atlas of developing human spinal cord**	5

Supplemental Tables	6

**Supplemental Table S1**	6

**Supplemental Table S2**	6

**Supplemental Table S3**	6

**Supplemental Table S4**	6

**Supplemental Table S5**	6

**Supplemental Table S6**	7

**Supplemental Table S7**	7

**Supplemental Table S8**	7

**Supplemental Table S9**	7

**Supplemental Table S10**	7

**Supplemental Table S11**	8

**Supplemental Table S12**	9

Supplemental Files	10

Supplemental Figures	11

**Supplemental Figure S1**	11

**Supplemental Figure S2**	13

**Supplemental Figure S3**	14

**Supplemental Figure S4**	16

**Supplemental Figure S5**	17

**Supplemental Figure S6**	18

**Supplemental Figure S7**	19

**Supplemental Figure S8**	20

**Supplemental Figure S9**	21

**Supplemental Figure S10**	22

# Supplemental Methods

*Neuroepithelial datasets*

## **Human neural stem cells**

The 10X Cell Ranger output for 3,049 U5-hNSCs (O’Connor et al. 2021) (available on NCBI GEO as GSM3267241 from GSE117003) was loaded into Seurat. Standard Seurat filters were applied requiring that the cells had to have at least 200 features per cell, and transcripts need to be expressed in at least three cells. Then the cells were further filtered to 3,035 cells by requiring the number of UMIs per cell to fall within the range of 1,000 to 20,000 UMIs, and the mitochondrial percentage of genes expressed per cell to fall within the range of 0.01 to 7%. For the purposes of these studies the 73 G1/other cells were removed because they did not align with any known cell cycle state. The 2,962 U5-hNSC cells were then normalized using SCTransform (Hafemeister and Satija 2019), principal components were calculated, and a UMAP was generated. Labels were transferred from O’Connor et al., 2021.

## **Glioblastoma tumor and cancer stem cells**

The 10X Cell Ranger outputs for two tumors (BT363 and BT368) and five cancer stem cell lines (BT322, BT324, BT333, BT363, and BT368) derived from tumors (available on the European Genome-Phenome Archive as EGAS00001004422) were loaded into Seurat. Standard Seurat filters were applied requiring that the cells had to have at least 200 features per cell, and transcripts need to be expressed in at least three cells. Each sample was further filtered by requiring the number of UMIs per cell to fall within a range of UMIs (**Supplemental Table S9**), and the mitochondrial percentage of genes expressed per cell to fall within a range of percents (**Supplemental Table S9**). The cells from each sample were then normalized using SCTransform (Hafemeister and Satija 2019), principal components were calculated, and a UMAP was generated. Clustering and expression of marker genes was used to remove non-tumor cell types from each sample:  oligodendrocytes - MBP and PLP1 (Valério-Gomes et al. 2018); astrocytes - ETNPPL (Zhang et al. 2016); neurons - RBFOX (Herculano-Houzel and Lent 2005); or immune cells - AIF1, CD14, CX3CR1, PTPRC. The number of cells for each sample are listed in the tumor cells column of Supplemental Table S8. The ccAFv2 classifier was applied to determine the cell cycle state of each cell.

## **Low grade glioma IDH mutant tumor cells**

The 10X Cell Ranger outputs for LGG275 (a grade 2 astrocytoma) (Augustus et al. 2021) and BT237 (a grade 3 oligodendroglioma) (now available on NCBI GEO as GSE263796 were loaded into Seurat. Standard Seurat filters were applied requiring that the cells had to have at least 200 features per cell, and transcripts need to be expressed in at least three cells. Each sample was further filtered by requiring the number of UMIs per cell to fall within a range of UMIs (**Supplemental Table S10**), and the mitochondrial percentage of genes expressed per cell to fall within a range of percents (**Supplemental Table S10**). The remaining cells were then normalized using SCTransform (Hafemeister and Satija 2019), principal components were calculated, and a UMAP was generated. The ccAFv2 classifier was applied to determine the cell cycle state of each cell.

## **Human neural stem cells from fetal tissue at post-conception weeks 3-12**

The data for the human gastrulation and early brain development scRNA-seq atlas from Zeng et al., 2023 (Zeng et al. 2023) (available from NCBI GEO as GSE155121) was downloaded as an h5ad file. The cells were subset to only the hNSCs by filtering the ‘Main Cell Type’, which is a metadata column in the h5ad object, for the keyword 'NSC' using the Python package scanpy (Wolf et al. 2018). Next, the 109,578 hNSCs were loaded into Seurat. Standard Seurat filters were applied requiring that the cells had to have at least 200 features per cell, and transcripts need to be expressed in at least three cells. Then the cells were subset by ‘week stage’ and filtered by requiring the number of UMIs per cell to fall within a range of UMIs (**Supplemental Table S11**), and the mitochondrial percentage of genes expressed per cell to fall within a range of percents (**Supplemental Table S11**). The remaining cells were then normalized using SCTransform (Hafemeister and Satija 2019) and the ccAFv2 classifier was applied to determine the cell cycle state of each cell. 

## **Human cells from fetal tissue at post-conception weeks 3-12**

The data for the human gastrulation and early brain development scRNA-seq atlas from Zeng et al., 2023 (Zeng et al. 2023) (available from NCBI GEO as GSE155121) was downloaded as an h5ad file. The cells were subset to each ‘Main Cell Type’, which is a metadata column in the h5ad object, using the Python package scanpy (Wolf et al. 2018) and saved as individual h5ad files. Data were loaded into Seurat. Standard Seurat filters were applied requiring that the cells had to have at least 200 features per cell, and transcripts need to be expressed in at least three cells. Then the cells were subset by ‘week stage’ and filtered by requiring the number of UMIs per cell to fall within a range of UMIs, and the mitochondrial percentage of genes expressed per cell to fall within a range of percents. The remaining cells were then normalized using SCTransform (Hafemeister and Satija 2019) and the ccAFv2 classifier was applied to determine the cell cycle state of each cell. Positive marker genes (log2 fold change ≥ 0.25; adjujsted p-value ≤ 0.05) were identified for each cell cycle state using the FindAllMarkers function. Neural G0 markers were tabulated among each dataset and across all datasets to identify common Neural G0 marker genes.

## **Atlas of the developing human telencephalon**

The processed data for the 4,261 cells from the Nowakowski et al., 2017 (Nowakowski et al. 2017) scRNA-seq atlas of the developing human telencephalon was downloaded from [http://bit.ly/cortexSingleCell](http://bit.ly/cortexSingleCell) and loaded into Seurat. The ccAFv2 classifier was applied to determine the cell cycle state of each cell, without running SCTransform.

## **Atlas of adult neurogenesis in the ventricular-subventricular zone (V-SVZ)**

The processed and SCTransformed scRNA-seq data for 24,261 cells from the ventricular-subventricular zone (V-SVZ) of adult mice from Cebrian-Silla et al., 2021 (Cebrian-Silla et al. 2021) was loaded into Seurat as an RDS file (available from GSM5039270 from NCBI GEO GSE165554). The ccAFv2 classifier was applied to determine the cell cycle state of each cell.

## **Activated and quiescent neural stem cells**

The processed data for the 129 cells from the Llorens-Bobadilla et al., 2015 (Llorens-Bobadilla et al. 2015) scRNA-seq characterization of activated and quiescent NSCs was loaded into Seurat as an expression matrix (available from NCBI GEO GSE67833). In lieu of SCTransform, the data was log-normalized, 3,000 variable features were detected, and the data was then scaled. The ccAFv2 classifier was applied to determine the cell cycle state of each cell, without running SCTransform.

The raw count matrix for the 279 cells from the Dulken et al., 2017 (Dulken et al. 2017) (available as NCBI BioProject PRJNA324289) were loaded into Seurat. Standard Seurat filters were applied requiring that the cells had to have at least 200 features per cell, and transcripts need to be expressed in at least three cells. The remaining cells were then normalized using SCTransform (Hafemeister and Satija 2019). The ccAFv2 classifier was applied to determine the cell cycle state of each cell.

## **Atlas of developing human spinal cord**

The 10X Cell Ranger outputs for eleven samples with both scRNA-seq and snRNA-seq from an atlas of the developing human spinal cord by Zhang et al., 2021 (Zhang et al. 2021) across 8 to 23 weeks post-conception (8 – 23 PCW; available on NCBI GEO as GSE136719) were loaded into Seurat. Standard Seurat filters were applied requiring that the cells/nuclei had to have at least 200 features per cell/nuclei, and transcripts need to be expressed in at least three cells/nuclei. Each sample was further filtered by requiring the number of UMIs per cell/nuclei to fall within a range of UMIs in the table below (**Supplemental Table S12**), and the mitochondrial percentage of genes expressed per cell to fall within a range of percents or for nuclei the counts per nuclei within a range of counts (**Supplemental Table S12**). The cells/nuclei from each sample were then normalized using SCTransform (Hafemeister and Satija 2019), principal components were calculated, and a UMAP was generated. The ccAFv2 classifier was applied to determine the cell cycle state of each cell.

# Supplemental Tables

**Supplemental Table S1**. Marker genes for each cell cycle state for U5-hNSCs.

[Supplemental Table S1 provided as a separate file]

**Supplemental Table S2**. Optimizing number of neurons in hidden layers.

[Supplemental Table S2 provided as a separate file]

**Supplemental Table S3**. Existing cell cycle classifiers.

| **Package** | **Phases predicted** | **Model** | **Underlying data** | **Reference** |
| --- | --- | --- | --- | --- |
| ccAF | Neural G0, G1, Late G1, S, S/G2, G2/M, M/Early G1 | Neural network | Human neural stem cells | **Our work**:  O’Connor et al, 2021 |
| Seurat/Scanpy | G1, S, G2/M | Gene scoring | Mouse hematopoietic progenitors | Butler et al, 2018 |
| tricycle | G1/G0, S, G2/M, M | Transfer learning | Mouse cortical neurospheres | Zheng et al, 2022 |
| SchwabeCC / Revelio | G1.S, S, G2, G2.M, M.G1 | PCA | mNeurosphere | Schwabe et al, 2020 |
| reCAT | G1, G1S, S, G2, G2M, M | Consensus traveling salesman problem and hidden Markov | Mouse embryonic stem cells | Liu et al, 2017 |
| peco | G1/G0, S, G2/M, M | Partition around medoids | Induced pluripotent stem cells | Hsiao et al, 2020 |
| cyclone | G1, S, G2M | Pairs method | Mouse embryonic stem cells | Scialdone et al, 2015 |

**Supplemental Table S4**. Likelihood threshold analysis for GSE155121.

[Supplemental Table S4 provided as a separate file]

**Supplemental Table S5**. Missing genes analysis for U5-hNSCs and PCW8 hNSCs.

[Supplemental Table S5 provided as a separate file]

**Supplemental Table S6**. Missing genes analysis for U5-hNSCs broken down by cell cycle state.

[Supplemental Table S6 provided as a separate file]

**Supplemental Table S7**. ccAFv2 regression analysis.

[Supplemental Table S7 provided as a separate file]

**Supplemental Table S8**. Nowakowski et al 2017 ccAF and ccAFv2 predictions.

[Supplemental Table S8 provided as a separate file]

**Supplemental Table S9****.** Glioblastoma tumor and cancer stem cells QC filters.

| **Sample** | **UMI bot.** | **UMI top** | **Mito. % bot.** | **Mito. % top** | **Resulting number of cells** | **Tumor cells** |
| --- | --- | --- | --- | --- | --- | --- |
| BT363 (Tumor) | 5,000 | 40,000 | 0.1 | 8 | 5,128 | 4,365 |
| BT368 (Tumor) | 6,000 | 50,000 | 0.1 | 8 | 1,556 | 1,262 |
| BT322 | 4,000 | 62,000 | 0.9 | 10 | 3,409 | 3,409 |
| BT324 | 5,000 | 40,000 | 0.9 | 6 | 4,377 | 3,351 |
| BT333 | 10,000 | 90,000 | 0.9 | 8 | 2,457 | 2,457 |
| BT363 | 5,000 | 80,000 | 0.9 | 10 | 4,917 | 4,770 |
| BT368 | 3,000 | 35,000 | 0.9 | 8 | 4,535 | 3,672 |

**Supplemental Table S10****.** Low grade glioma IDH mutant tumor cells QC filters.

| **Sample** | **UMI bot.** | **UMI top** | **Mito. % bot.** | **Mito. % top** | **Resulting number of cells** |
| --- | --- | --- | --- | --- | --- |
| LGG275_GF | 5,000 | 76,000 | 0.1 | 15 | 2,232 |
| LGG275_noGF | 6,000 | 35,000 | 0.1 | 8 | 1,744 |
| BT237_GF | 6,000 | 90,000 | 1 | 15 | 1,485 |
| BT237_noGF | 6,000 | 90,000 | 3 | 17 | 3,074 |

**Supplemental Table S11****.** Human neural stem cells from fetal tissues at post-conception weeks 3-12 QC filters.

| **Sample** | **UMI bot.** | **UMI top** | **Mito. % bot.** | **Mito. % top** | **Resulting number of cells** |
| --- | --- | --- | --- | --- | --- |
| PCW3-1 | 2,000 | 40,000 | 0.1 | 4 | 1,177 |
| PCW4-1 | 4,000 | 40,000 | 0.1 | 8 | 5,297 |
| PCW4-2 | 4,000 | 32,000 | 0.1 | 6 | 12,458 |
| PCW4-3 | 4,000 | 28,000 | 0.1 | 6 | 12,269 |
| PCW5-1 | 4,000 | 32,000 | 0.1 | 5 | 6,126 |
| PCW5-2 | 4,000 | 30,000 | 0.5 | 14 | 10,009 |
| PCW5-3 | 4,000 | 35,000 | 0.5 | 6 | 9,133 |
| PCW6-1 | 4,000 | 32,000 | 0.1 | 4 | 6,040 |
| PCW7-1 | 4,000 | 30,000 | 0.1 | 5 | 15,246 |
| PCW8-1 | 4,000 | 23,000 | 0.1 | 4 | 2,562 |
| PCW9-1 | 4,000 | 23,000 | 0.1 | 4 | 5,575 |
| PCW9-2 | 4,000 | 25,000 | 0.1 | 5 | 4,277 |
| PCW12-1 | 4,000 | 20,000 | 0.1 | 4 | 4,128 |

**Supplemental Table S12****.** Atlas of developing human spinal cord QC filters. 

| **Sample** | **Location** | **Cells or Nuclei** | **Raw cells** | **UMI bot.** | **UMI top** | **Mito. % or counts bot.** | **Mito. % or count top** | **Resulting number of cells** |
| --- | --- | --- | --- | --- | --- | --- | --- | --- |
| GW8 | Spinal Whole | Cells | 24,874 | 3,000 | 50,000 | 0.1 | 8 | 11,047 |
| GW8 | Spinal Whole | Nuclei | 25,282 | 200 | 15,000 | 200 | 6,000 | 25,269 |
| GW10 | Cervical | Cells | 13,907 | 3,000 | 50,000 | 0.1 | 8 | 5,763 |
| GW10 | Cervical | Nuclei | 7,509 | 200 | 55,000 | 200 | 10,000 | 7,485 |
| GW10 | Lumbar | Cells | 10,456 | 3,000 | 45,000 | 0.1 | 8 | 4,695 |
| GW10 | Lumbar | Nuclei | 19,887 | 200 | 70,000 | 200 | 11,000 | 19,869 |
| GW10 | Thoracic | Cells | 11,229 | 3,000 | 40,000 | 0.1 | 8 | 5,307 |
| GW10 | Thoracic | Nuclei | 7,026 | 200 | 55,000 | 200 | 10,000 | 7,011 |
| GW11 | Cervical | Cells | 18,691 | 3,000 | 50,000 | 0.1 | 5 | 11,377 |
| GW11 | Cervical | Nuclei | 9,069 | 200 | 15,000 | 200 | 5,500 | 9,067 |
| GW11 | Lumbar | Cells | 8,099 | 2,000 | 18,000 | 0 | 5 | 500 |
| GW11 | Lumbar | Nuclei | 8,099 | 200 | 15,000 | 200 | 6,000 | 8,094 |
| GW11 | Thoracic | Cells | 8,026 | 2,000 | 12,000 | 0 | 10 | 1,122 |
| GW11 | Thoracic | Nuclei | 17,763 | 200 | 30,000 | 200 | 6,000 | 17,676 |
| GW20 | Cervical | Cells | 11,182 | 3,000 | 30,000 | 0.1 | 8 | 9,441 |
| GW20 | Cervical | Nuclei | 10,146 | 200 | 18,000 | 200 | 6,000 | 10,137 |
| GW20 | Lumbar | Cells | 12,189 | 3,000 | 40,000 | 0.1 | 10 | 9,011 |
| GW20 | Lumbar | Nuclei | 8,493 | 200 | 16,000 | 200 | 6,000 | 8,484 |
| GW20 | Thoracic | Cells | 11,745 | 3,000 | 25,000 | 0.8 | 8 | 9,694 |
| GW20 | Thoracic | Nuclei | 10,004 | 200 | 12,000 | 200 | 5,000 | 9,999 |
| GW23 | Spinal Whole | Cells | 11,293 | 3,000 | 28,000 | 0.8 | 10 | 8,602 |
| GW23 | Spinal Whole | Nuclei | 11,293 | 200 | 28,000 | 200 | 5,000 | 11,217 |

# Supplemental Files

1. Supplementary_Code.tgz – This file contains the code for running data and analysis pipelines for producing paper figures. It can also be found at [https://github.com/plaisier-lab/ccAFv2/tree/main/code](https://github.com/plaisier-lab/ccAFv2/tree/main/code). The data can be found at [https://zenodo.org/doi/10.5281/zenodo.10963136](https://zenodo.org/doi/10.5281/zenodo.10963136). 

# Supplemental Figures

**Supplemental Figure S1****.** Building ccAFv2 and comparison of cell labels and ccAFv2 cell cycle state classifications. **A.** Pipeline for the quality control, training, and evaluation of the ccAFv2 classifier. Data is quality controlled and normalized using sctransform. Then the data is split 80% for training and 20% for testing using 10-fold cross validation. For each iteration three steps are performed:  1) the ANN is trained using the training dataset, 2) then the trained classifier is used to predict on the test set, and 3) the predicted labels are compared to the true labels to evaluate the accuracy of the classifier. **B.** Overlay of cell labels (O’Connor et al. 2021) and ccAFv2 predictions on the U5-hNSCs UMAP. **C.** Cell fractions of U5‐hNSC defined cell labels colorized by the ccAFv2 predictions. **D**. Heatmap showing the expression levels of the top three important features for each ccAFv2 state. Data is grouped by ccAFv2-classified *in vivo* human neural stem cells collected at nine weeks post-conception (PCW 9-1) (Zeng et al, 2023) 

**Supplemental Figure S2****.** Application of ccAFv2 to hNSC in vivo and determining the optimal likelihood threshold. **A**. Cells predicted tabulated from ccAFv2 application with likelihood thresholds 0.0 to 0.9 to hNSCs from 3 – 12 PCW fetal tissue (Zeng et al. 2023). Dashed line indicates median cells predicted across all week stages. **B**. Adjusted mutual information (AMI) scores for ccAFv2 cell cycle predictions relative to the ccSeurat cell cycle states tabulated from ccAFv2 application with likelihood thresholds 0.0 to 0.9 to hNSCs from 3 – 12 PCW fetal tissue. Dashed line indicates median AMI across all week stages. Red dashed line indicates significantly improved median AMI score due to applying threshold.

**Supplemental Figure S3****.** U5-hNSC missing gene analyses. **A**. Sensitivity analysis shows how a random sampling of 10-99% information from the U5-hNSCs effects the error rate of the ccAFv2 classifier with a likelihood threshold of 0.5. **B**. Error rate in (**A**) broken down by cell cycle state. **C**. Percentage of cells predicted for U5-hNSC data broken down by cell cycle state and percentage of missing information. **D**. Sensitivity analysis shows how a random sampling of 10-99% information from the U5-hNSCs effects the error rate of the ccAFv2 classifier with a likelihood threshold of 0.7, and **E**. 0.9. **F**. ccAFv2 threshold plot for U5-hNSCs. 

**Supplemental Figure S4****.** hNSCs from PCW8 missing gene analyses. **A.** Sensitivity analysis shows how a random sampling of 10-99% information from the PCW8-derived hNSCs from Zeng et al, 2023 effects the AMI of the ccAFv2 classifier relative to the ccSeurat predictions with a likelihood threshold of 0.5, **B.** 0.7, and **C.** 0.9.

**Supplemental Figure S5****.** Application of ccAFv2 to low-grade glioma scRNA-seq samples grown with and without growth factor. **A.** Cell fractions of a grade 2 astrocytoma (LGG275) ccAFv2 cell cycle classifications using likelihood thresholds 0.0 to 0.9. G = cells grown with growth factor; N = cells grown without growth factor. **B.** Cell fractions of a grade 3 oligodendroglioma (BT237) ccAFv2 cell cycle classifications using likelihood thresholds 0.0 to 0.9. G = cells grown with growth factor; N = cells grown without growth factor.  

 

**Supplemental Figure S6****.** Cell cycle regression. **A.** U5-hNSC PCA plot colorized by ccAFv2. **B.** U5-hNSC with S and G2/M ccAFv2 classes regressed PCA plot colorized by ccAFv2. **C** U5-hNSC with all cycling ccAFv2 classed regressed PCA plot colorized by ccAFv2. **D**. U5-hNSC PCA plot colorized by ccSeurat. **E**. U5-hNSC with S and G2/M ccSeurat classes regressed PCA plot colorized by ccSeurat. **F**. Venn diagram of ccAFv2 cell cycle state marker genes and ccSeurat cell cycle state marker genes.

**Supplemental Figure S7****.** Application of ccAFv2 to quiescent (qNSC) and activated (aNSC) neural stem cells from Llorens‐Bobadilla et al, 2015. **A.** Cell fractions of qNSC1 ccAFv2 cell cycle classifications using likelihood thresholds 0.0 to 0.9. **B.** Cell fractions of qNSC2 ccAFv2 cell cycle classifications using likelihood thresholds 0.0 to 0.9. **C.** Cell fractions of aNSC1 ccAFv2 cell cycle classifications using likelihood thresholds 0.0 to 0.9. **D.** Cell fractions of aNSC2 ccAFv2 cell cycle classifications using likelihood thresholds 0.0 to 0.9.

**Supplemental Figure S8****.** Validation of cell cycle states in U5-hNSC data using QuieScore and cyclin expression levels. **A**. Application of QuieScore to the U5-hNSCs (q_score_raw > 3 defined G0 cells). **B**. Hypergeometric enrichment of QuieScore and U5-hNSC cell labels (O’Connor et al, 2021). Values reported are the negative log10 of the p-values.

**Supplemental Figure S9****.** Expression of skin and bone marker genes to the developing cortex of a male C57BL/6 mouse embryo at E15.5. **A**. H&E staining for the developing embryo cortex. **B**-**C**. Expression of skin (Krt5) and bone (Col1a1) key marker genes in the developing embryo cortex.

**Supplemental Figure S10****.** Application of the R and python-based ccAFv2. **A.** Cell percentages of *in vivo* hNSCs from PCW8 from Zeng et al, 2023 of ccAFv2 cell cycle state classifications using the R package Seurat v4 and v5, and Python package Scanpy.  

**References**

Augustus M, Pineau D, Aimond F, Azar S, Lecca D, Scamps F, Muxel S, Darlix A, Ritchie W, Gozé C, et al. 2021. Identification of CRYAB+ KCNN3+ SOX9+ Astrocyte-Like and EGFR+ PDGFRA+ OLIG1+ Oligodendrocyte-Like Tumoral Cells in Diffuse IDH1-Mutant Gliomas and Implication of NOTCH1 Signalling in Their Genesis. *Cancers* **13**: 2107.

Cebrian-Silla A, Nascimento MA, Redmond SA, Mansky B, Wu D, Obernier K, Romero Rodriguez R, Gonzalez-Granero S, García-Verdugo JM, Lim DA, et al. 2021. Single-cell analysis of the ventricular-subventricular zone reveals signatures of dorsal and ventral adult neurogenesis eds. J.G. Gleeson, M.E. Bronner, and D. Van der Kooy. *eLife* **10**: e67436.

Dulken BW, Leeman DS, Boutet SC, Hebestreit K, Brunet A. 2017. Single-Cell Transcriptomic Analysis Defines Heterogeneity and Transcriptional Dynamics in the Adult Neural Stem Cell Lineage. *Cell Rep* **18**: 777–790.

Hafemeister C, Satija R. 2019. Normalization and variance stabilization of single-cell RNA-seq data using regularized negative binomial regression. *Genome Biol* **20**: 296.

Herculano-Houzel S, Lent R. 2005. Isotropic Fractionator: A Simple, Rapid Method for the Quantification of Total Cell and Neuron Numbers in the Brain. *J Neurosci* **25**: 2518–2521.

Llorens-Bobadilla E, Zhao S, Baser A, Saiz-Castro G, Zwadlo K, Martin-Villalba A. 2015. Single-Cell Transcriptomics Reveals a Population of Dormant Neural Stem Cells that Become Activated upon Brain Injury. *Cell Stem Cell* **17**: 329–340.

Nowakowski TJ, Bhaduri A, Pollen AA, Alvarado B, Mostajo-Radji MA, Di Lullo E, Haeussler M, Sandoval-Espinosa C, Liu SJ, Velmeshev D, et al. 2017. Spatiotemporal gene expression trajectories reveal developmental hierarchies of the human cortex. *Science* **358**: 1318–1323.

O’Connor SA, Feldman HM, Arora S, Hoellerbauer P, Toledo CM, Corrin P, Carter L, Kufeld M, Bolouri H, Basom R, et al. 2021. Neural G0: a quiescent-like state found in neuroepithelial-derived cells and glioma. *Mol Syst Biol* **17**: e9522.

Valério-Gomes B, Guimarães DM, Szczupak D, Lent R. 2018. The Absolute Number of Oligodendrocytes in the Adult Mouse Brain. *Front Neuroanat* **12**: 90.

Wolf FA, Angerer P, Theis FJ. 2018. SCANPY: large-scale single-cell gene expression data analysis. *Genome Biol* **19**: 15.

Zeng B, Liu Z, Lu Y, Zhong S, Qin S, Huang L, Zeng Y, Li Z, Dong H, Shi Y, et al. 2023. The single-cell and spatial transcriptional landscape of human gastrulation and early brain development. *Cell Stem Cell* **30**: 851-866.e7.

Zhang Q, Wu X, Fan Y, Jiang P, Zhao Y, Yang Y, Han S, Xu B, Chen B, Han J, et al. 2021. Single‐cell analysis reveals dynamic changes of neural cells in developing human spinal cord. *EMBO Rep* **22**: e52728.

Zhang Y, Sloan SA, Clarke LE, Caneda C, Plaza CA, Blumenthal PD, Vogel H, Steinberg GK, Edwards MSB, Li G, et al. 2016. Purification and characterization of progenitor and mature human astrocytes reveals transcriptional and functional differences with mouse. *Neuron* **89**: 37–53.
