# Supplementary material for: Breaking out of the cycle: Including quiescence in cell cycle classification
Source: bioRxiv. 2024 Apr 20:2024.04.16.589816. Preprint. [Version 1] doi: 10.1101/2024.04.16.589816 (PMC11042294; doi:10.1101/2024.04.16.589816)
Supplement: Supplementary file 1 [file media-1.docx]

**Supplementary information for Breaking out of the cycle:  Including quiescence in cell cycle classification**

Samantha A. O’Connor^1^, Leonor Garcia ^2^, Anoop P. Patel ^3,4^, Jean-Philippe Hugnot ^2^, Patrick J. Paddison ^5^, Christopher L. Plaisier ^1,^*

^1 ^School of Biological and Health Systems Engineering, Arizona State University, Tempe AZ, USA

^2^ Institut de Génomique Fonctionnelle, Université de Montpellier, CNRS, INSERM, 141 rue de la Cardonille, 34091, Montpellier, France.

^3^ Brotman-Baty Institute for Precision Medicine, University of Washington, Seattle, WA, USA.

^4 ^Department of Neurosurgery, Preston Robert Tisch Brain Tumor Center, Duke University, Durham, NC, USA.

^5^ Human Biology Division, Fred Hutchinson Cancer Research Center, Seattle WA, USA

* To whom correspondence should be addressed: Christopher Plaisier, E-mail:  [plaisier@asu.edu](mailto:plaisier@asu.edu), Phone: (480) 965-6832, Address:  P.O. Box 879709, Tempe, AZ 87287-9709

Table of Contents 

Supplementary Methods	2

**Human neural stem cells**	2

**Glioblastoma tumor and cancer stem cells**	2

**Low grade glioma IDH mutant tumor cells**	2

**Human neural stem cells from fetal tissue at post-conception weeks 3-12**	3

**Human cells from fetal tissue at post-conception week 4**	3

**Spatial transcriptomics of a human fetus at 4 weeks post-conception**	3

**Atlas of the developing human telencephalon**	3

**Atlas of adult neurogenesis in the ventricular-subventricular zone (V-SVZ)**	3

**Activated and quiescent neural stem cells**	3

**Atlas of developing human spinal cord**	4

Supplementary Tables	5

**Supplementary Table S1**	5

**Supplementary Table S2**	5

**Supplementary Table S3**	5

**Supplementary Table S4**	5

**Supplementary Table S5**	5

**Supplementary Table S6**	6

**Supplementary Table S7**	6

**Supplementary Table S9**	6

**Supplementary Table S10**	7

**Supplementary Table S11**	7

**Supplementary Table S12**	8

Supplementary Figures	9

**Supplementary Figure S1**	9

**Supplementary Figure S2**	10

**Supplementary Figure S3**	11

**Supplementary Figure S4**	12

**Supplementary Figure S5**	13

**Supplementary Figure S6**	14

**Supplementary Figure S7**	15

**Supplementary Figure S8**	16

# Supplementary Methods

*Neuroepithelial datasets*

## **Human neural stem cells**

The 10X Cell Ranger output for 3,049 U5-hNSCs (O’Connor et al. 2021) (available on NCBI GEO as GSM3267241 from GSE117003) was loaded into Seurat. Standard Seurat filters were applied requiring that the cells had to have at least 200 features per cell, and transcripts need to be expressed in at least three cells. Then the cells were further filtered to 3,035 cells by requiring the number of UMIs per cell to fall within the range of 1,000 to 20,000 UMIs, and the mitochondrial percentage of genes expressed per cell to fall within the range of 0.01 to 7%. For the purposes of these studies the 73 G1/other cells were removed because they did not align with any known cell cycle state. The 2,962 U5-hNSC cells were then normalized using SCTransform (Hafemeister and Satija 2019), principal components were calculated, and a UMAP was generated. Labels were transferred from O’Connor et al., 2021.

## **Glioblastoma tumor and cancer stem cells**

The 10X Cell Ranger outputs for two tumors (BT363 and BT368) and five cancer stem cell lines (BT322, BT324, BT333, BT363, and BT368) derived from tumors (available on the European Genome-Phenome Archive as EGAS00001004422) were loaded into Seurat. Standard Seurat filters were applied requiring that the cells had to have at least 200 features per cell, and transcripts need to be expressed in at least three cells. Each sample was further filtered by requiring the number of UMIs per cell to fall within a range of UMIs (**Supplementary Table S8**), and the mitochondrial percentage of genes expressed per cell to fall within a range of percents (**Supplementary Table S8**). The cells from each sample were then normalized using SCTransform (Hafemeister and Satija 2019), principal components were calculated, and a UMAP was generated. Clustering and expression of marker genes was used to remove non-tumor cell types from each sample:  oligodendrocytes - MBP and PLP1 (Valério-Gomes et al. 2018); astrocytes - ETNPPL (Zhang et al. 2016); neurons - RBFOX (Herculano-Houzel and Lent 2005); or immune cells - AIF1, CD14, CX3CR1, PTPRC. The number of cells for each sample are listed in the tumor cells column of Supplementary Table S8. The ccAFv2 classifier was applied to determine the cell cycle state of each cell.

## **Low grade glioma IDH mutant tumor cells**

The 10X Cell Ranger outputs for LGG275 (a grade 2 astrocytoma) (Augustus et al. 2021) and BT237 (a grade 3 oligodendroglioma) (now available on NCBI GEO as GSE263796 were loaded into Seurat. Standard Seurat filters were applied requiring that the cells had to have at least 200 features per cell, and transcripts need to be expressed in at least three cells. Each sample was further filtered by requiring the number of UMIs per cell to fall within a range of UMIs (**Supplementary Table S9**), and the mitochondrial percentage of genes expressed per cell to fall within a range of percents (**Supplementary Table S9**). The remaining cells were then normalized using SCTransform (Hafemeister and Satija 2019), principal components were calculated, and a UMAP was generated. The ccAFv2 classifier was applied to determine the cell cycle state of each cell.

## **Human neural stem cells from fetal tissue at post-conception weeks 3-12**

The data for the human gastrulation and early brain development scRNA-seq atlas from Zeng et al., 2023 (Zeng et al. 2023) (available from NCBI GEO as GSE155121) was downloaded as an h5ad file. The cells were subset to only the hNSCs by filtering the ‘Main Cell Type’ for the keyword 'NSC' using the Python package scanpy (Wolf et al. 2018). Next, the 109,578 hNSCs were loaded into Seurat. Standard Seurat filters were applied requiring that the cells had to have at least 200 features per cell, and transcripts need to be expressed in at least three cells. Then the cells were subset by ‘week stage’ and filtered by requiring the number of UMIs per cell to fall within a range of UMIs (**Supplementary Table S10**), and the mitochondrial percentage of genes expressed per cell to fall within a range of percents (**Supplementary Table S10**). The remaining cells were then normalized using SCTransform (Hafemeister and Satija 2019) and the ccAFv2 classifier was applied to determine the cell cycle state of each cell. 

## **Human cells from fetal tissue at post-conception week 4**

The data for the human gastrulation and early brain development scRNA-seq atlas from Zeng et al., 2023 (Zeng et al. 2023) (available from NCBI GEO as GSE155121) was downloaded as an h5ad file. The cells were subset to only the GW4-1 cells by filtering the ‘week stage’ for the keyword 'W4-1' using the Python package scanpy (Wolf et al. 2018). This includes all cells (i.e., is not limited to hNSCs). Next, the 32,250 PCW4 cells were loaded into Seurat. Standard Seurat filters were applied requiring that the cells had to have at least 200 features per cell, and transcripts need to be expressed in at least three cells. Then the cells were further filtered to 27,380 cells by requiring the number of UMIs per cell to fall within the range of 3,000 to 50,000 UMIs, and the mitochondrial percentage of genes expressed per cell to fall within the range of 1 to 12%. The remaining cells were then normalized using SCTransform (Hafemeister and Satija 2019) and the ccAFv2 classifier was applied to determine the cell cycle state of each cell.

## **Spatial transcriptomics of a human fetus at 4 weeks post-conception**

The 10X Genomics Visium spatial transcriptomics data for eight slices from a human fetus at 4 weeks post-conception from the human gastrulation and early brain development scRNA-seq atlas from Zeng et al., 2023 (Zeng et al. 2023) (available from NCBI GEO as GSE155121) were downloaded as RData files. The Seurat objects loaded from the RData files were already quality controlled and SCTransformed. This allowed ccAFv2 to be directly applied to determine the cell cycle states and likelihood of each state for every spot characterized by ST-seq. The number of spots for each slice is listed in the supplementary table below (**Supplementary Table S11**).

## **Atlas of the developing human telencephalon**

The processed data for the 4,261 cells from the Nowakowski et al., 2017 (Nowakowski et al. 2017) scRNA-seq atlas of the developing human telencephalon was downloaded from [http://bit.ly/cortexSingleCell](http://bit.ly/cortexSingleCell) and loaded into Seurat. The ccAFv2 classifier was applied to determine the cell cycle state of each cell, without running SCTransform.

## **Atlas of adult neurogenesis in the ventricular-subventricular zone (V-SVZ)**

The processed and SCTransformed scRNA-seq data for 24,261 cells from the ventricular-subventricular zone (V-SVZ) of adult mice from Cebrian-Silla et al., 2021 (Cebrian-Silla et al. 2021) was loaded into Seurat as an RDS file (available from GSM5039270 from NCBI GEO GSE165554). The ccAFv2 classifier was applied to determine the cell cycle state of each cell.

## **Activated and quiescent neural stem cells**

The processed data for the 129 cells from the Llorens-Bobadilla et al., 2015 (Llorens-Bobadilla et al. 2015) scRNA-seq characterization of activated and quiescent NSCs was loaded into Seurat as an expression matrix (available from NCBI GEO GSE67833). In lieu of SCTransform, the data was log-normalized, 3,000 variable features were detected, and the data was then scaled. The ccAFv2 classifier was applied to determine the cell cycle state of each cell, without running SCTransform.

The raw count matrix for the 279 cells from the Dulken et al., 2017 (Dulken et al. 2017) (available as NCBI BioProject PRJNA324289) were loaded into Seurat. Standard Seurat filters were applied requiring that the cells had to have at least 200 features per cell, and transcripts need to be expressed in at least three cells. The remaining cells were then normalized using SCTransform (Hafemeister and Satija 2019). The ccAFv2 classifier was applied to determine the cell cycle state of each cell.

## **Atlas of developing human spinal cord**

The 10X Cell Ranger outputs for eleven samples with both scRNA-seq and snRNA-seq from an atlas of the developing human spinal cord by Zhang et al., 2021 (Zhang et al. 2021) across 8 to 23 weeks post-conception (8 – 23 PCW; available on NCBI GEO as GSE136719) were loaded into Seurat. Standard Seurat filters were applied requiring that the cells/nuclei had to have at least 200 features per cell/nuclei, and transcripts need to be expressed in at least three cells/nuclei. Each sample was further filtered by requiring the number of UMIs per cell/nuclei to fall within a range of UMIs in the table below (**Supplementary Table S12**), and the mitochondrial percentage of genes expressed per cell to fall within a range of percents or for nuclei the counts per nuclei within a range of counts (**Supplementary Table S12**). The cells/nuclei from each sample were then normalized using SCTransform (Hafemeister and Satija 2019), principal components were calculated, and a UMAP was generated. The ccAFv2 classifier was applied to determine the cell cycle state of each cell.

# Supplementary Tables

**Supplementary Table S1**. Marker genes for each cell cycle state for U5-hNSCs.

[Supplementary Table S1 provided as a separate file]

**Supplementary Table S2**. Optimizing number of neurons in hidden layers.

[Supplementary Table S2 provided as a separate file]

**Supplementary Table S3**. Existing cell cycle classifiers.

| **Package** | **Phases predicted** | **Model** | **Underlying data** | **Reference** |
| --- | --- | --- | --- | --- |
| ccAF | Neural G0, G1, Late G1, S, S/G2, G2/M, M/Early G1 | Neural network | Human neural stem cells | **Our work**:  O’Connor et al, 2021 |
| Seurat/Scanpy | G1, S, G2/M | Gene scoring | Mouse hematopoietic progenitors | Butler et al, 2018 |
| tricycle | G1/G0, S, G2/M, M | Transfer learning | Mouse cortical neurospheres | Zheng et al, 2022 |
| SchwabeCC / Revelio | G1.S, S, G2, G2.M, M.G1 | PCA | mNeurosphere | Schwabe et al, 2020 |
| reCAT | G1, G1S, S, G2, G2M, M | Consensus traveling salesman problem and hidden Markov | Mouse embryonic stem cells | Liu et al, 2017 |
| peco | G1/G0, S, G2/M, M | Partition around medoids | Induced pluripotent stem cells | Hsiao et al, 2020 |
| cyclone | G1, S, G2M | Pairs method | Mouse embryonic stem cells | Scialdone et al, 2015 |

**Supplementary Table S4**. Likelihood threshold analysis for GSE155121.

[Supplementary Table S4 provided as a separate file]

**Supplementary Table S5**. Missing genes analysis for U5-hNSCs and PCW8 hNSCs.

[Supplementary Table S5 provided as a separate file]

**Supplementary Table S6**. ccAFv2 regression analysis.

[Supplementary Table S6 provided as a separate file]

**Supplementary Table S7**. Nowakowski et al 2017 ccAF and ccAFv2 predictions.

[Supplementary Table S7 provided as a separate file]

**Supplementary Table S8.** Glioblastoma tumor and cancer stem cells QC filters.

| **Sample** | **UMI bot.** | **UMI top** | **Mito. % bot.** | **Mito. % top** | **Resulting number of cells** | **Tumor cells** |
| --- | --- | --- | --- | --- | --- | --- |
| BT363 (Tumor) | 5000 | 40000 | 0.1 | 8 | 5128 | 4365 |
| BT368 (Tumor) | 6000 | 50000 | 0.1 | 8 | 1556 | 1262 |
| BT322 | 4000 | 62000 | 0.9 | 10 | 3409 | 3409 |
| BT324 | 5000 | 40000 | 0.9 | 6 | 4377 | 3351 |
| BT333 | 10000 | 90000 | 0.9 | 8 | 2457 | 2457 |
| BT363 | 5000 | 80000 | 0.9 | 10 | 4917 | 4770 |
| BT368 | 3000 | 35000 | 0.9 | 8 | 4535 | 3672 |

**Supplementary Table S9****.** Low grade glioma IDH mutant tumor cells QC filters.

| **Sample** | **UMI bot.** | **UMI top** | **Mito. % bot.** | **Mito. % top** | **Resulting number of cells** |
| --- | --- | --- | --- | --- | --- |
| LGG275_GF | 5000 | 76000 | 0.1 | 15 | 2232 |
| LGG275_noGF | 6000 | 35000 | 0.1 | 8 | 1744 |
| BT237_GF | 6000 | 90000 | 1 | 15 | 1485 |
| BT237_noGF | 6000 | 90000 | 3 | 17 | 3074 |

**Supplementary Table S10****.** Human neural stem cells from fetal tissues at post-conception weeks 3-12 QC filters.

| **Sample** | **UMI bot.** | **UMI top** | **Mito. % bot.** | **Mito. % top** | **Resulting number of cells** |
| --- | --- | --- | --- | --- | --- |
| PCW3-1 | 2000 | 40000 | 0.1 | 4 | 1177 |
| PCW4-1 | 4000 | 40000 | 0.1 | 8 | 5297 |
| PCW4-2 | 4000 | 32000 | 0.1 | 6 | 12458 |
| PCW4-3 | 4000 | 28000 | 0.1 | 6 | 12269 |
| PCW5-1 | 4000 | 32000 | 0.1 | 5 | 6126 |
| PCW5-2 | 4000 | 30000 | 0.5 | 14 | 10009 |
| PCW5-3 | 4000 | 35000 | 0.5 | 6 | 9133 |
| PCW6-1 | 4000 | 32000 | 0.1 | 4 | 6040 |
| PCW7-1 | 4000 | 30000 | 0.1 | 5 | 15246 |
| PCW8-1 | 4000 | 23000 | 0.1 | 4 | 2562 |
| PCW9-1 | 4000 | 23000 | 0.1 | 4 | 5575 |
| PCW9-2 | 4000 | 25000 | 0.1 | 5 | 4277 |
| PCW12-1 | 4000 | 20000 | 0.1 | 4 | 4128 |

**Supplementary Table S11****.** Spatial transcriptomics of a human fetus at 4 weeks post-conception slices and number of spots. 

| **Sample** | **Slice** | **Resulting number of spots** |
| --- | --- | --- |
| PCW4 | Slice 1 | 1177 |
| PCW4 | Slice 2 | 5297 |
| PCW4 | Slice 3 | 12458 |
| PCW4 | Slice 4 | 12269 |
| PCW4 | Slice 5 | 6126 |
| PCW4 | Slice 6 | 10009 |
| PCW4 | Slice 7 | 9133 |
| PCW4 | Slice 8 | 6040 |

**Supplementary Table S12****.** Atlas of developing human spinal cord QC filters. 

| **Sample** | **Location** | **Cells or Nuclei** | **Raw cells** | **UMI bot.** | **UMI top** | **Mito. % or counts bot.** | **Mito. % or count top** | **Resulting number of cells** |
| --- | --- | --- | --- | --- | --- | --- | --- | --- |
| GW8 | Spinal Whole | Cells | 24874 | 3000 | 50000 | 0.1 | 8 | 11047 |
| GW8 | Spinal Whole | Nuclei | 25282 | 200 | 15000 | 200 | 6000 | 25269 |
| GW10 | Cervical | Cells | 13907 | 3000 | 50000 | 0.1 | 8 | 5763 |
| GW10 | Cervical | Nuclei | 7509 | 200 | 55000 | 200 | 10000 | 7485 |
| GW10 | Lumbar | Cells | 10456 | 3000 | 45000 | 0.1 | 8 | 4695 |
| GW10 | Lumbar | Nuclei | 19887 | 200 | 70000 | 200 | 11000 | 19869 |
| GW10 | Thoracic | Cells | 11229 | 3000 | 40000 | 0.1 | 8 | 5307 |
| GW10 | Thoracic | Nuclei | 7026 | 200 | 55000 | 200 | 10000 | 7011 |
| GW11 | Cervical | Cells | 18691 | 3000 | 50000 | 0.1 | 5 | 11377 |
| GW11 | Cervical | Nuclei | 9069 | 200 | 15000 | 200 | 5500 | 9067 |
| GW11 | Lumbar | Cells | 8099 | 2000 | 18000 | 0 | 5 | 500 |
| GW11 | Lumbar | Nuclei | 8099 | 200 | 15000 | 200 | 6000 | 8094 |
| GW11 | Thoracic | Cells | 8026 | 2000 | 12000 | 0 | 10 | 1122 |
| GW11 | Thoracic | Nuclei | 17763 | 200 | 30000 | 200 | 6000 | 17676 |
| GW20 | Cervical | Cells | 11182 | 3000 | 30000 | 0.1 | 8 | 9441 |
| GW20 | Cervical | Nuclei | 10146 | 200 | 18000 | 200 | 6000 | 10137 |
| GW20 | Lumbar | Cells | 12189 | 3000 | 40000 | 0.1 | 10 | 9011 |
| GW20 | Lumbar | Nuclei | 8493 | 200 | 16000 | 200 | 6000 | 8484 |
| GW20 | Thoracic | Cells | 11745 | 3000 | 25000 | 0.8 | 8 | 9694 |
| GW20 | Thoracic | Nuclei | 10004 | 200 | 12000 | 200 | 5000 | 9999 |
| GW23 | Spinal Whole | Cells | 11293 | 3000 | 28000 | 0.8 | 10 | 8602 |
| GW23 | Spinal Whole | Nuclei | 11293 | 200 | 28000 | 200 | 5000 | 11217 |

# Supplementary Figures

**Supplementary Figure S1****.** Comparison of cell labels and ccAFv2 cell cycle state classifications. **A.** Overlay of cell labels (O’Connor et al. 2021) and ccAFv2 predictions on the U5-hNSCs UMAP. **B.** Cell fractions of U5‐hNSC defined cell labels colorized by the ccAFv2 predictions. 

**Supplementary Figure S2****.** Application of ccAFv2 to hNSC in vivo and determining the optimal likelihood threshold. **A**. Cells predicted tabulated from ccAFv2 application with likelihood thresholds 0.0 to 0.9 to hNSCs from 3 – 12 PCW fetal tissue (Zeng et al. 2023). Dashed line indicates median cells predicted across all week stages. **B**. Adjusted mutual information (AMI) scores for ccAFv2 cell cycle predictions relative to the ccSeurat cell cycle states tabulated from ccAFv2 application with likelihood thresholds 0.0 to 0.9 to hNSCs from 3 – 12 PCW fetal tissue. Dashed line indicates median AMI across all week stages. Red dashed line indicates significantly improved median AMI score due to applying threshold.

**Supplementary Figure S3****.** U5-hNSC missing gene analyses. **A**. Sensitivity analysis shows how a random sampling of a percentage of the 861 ccAFv2 input genes (10-99%) from the U5-hNSCs effects the error rate of the ccAFv2 classifier with a likelihood threshold of 0.5, **B**. 0.7, and **C**. 0.9.

**Supplementary Figure S4****.** hNSCs from PCW8 missing gene analyses. **A.** Sensitivity analysis shows how a random sampling of a percentage of the 861 ccAFv2 input genes (10-99%) from the PCW8-derived hNSCs from Zeng et al, 2023 effects the AMI of the ccAFv2 classifier relative to the ccSeurat predictions with a likelihood threshold of 0.5, **B.** 0.7, and **C.** 0.9.

**Supplementary Figure S5****.** Application of ccAFv2 to low-grade glioma scRNA-seq samples grown with and without growth factor. **A.** Cell fractions of a grade 2 astrocytoma (LGG275) ccAFv2 cell cycle classifications using likelihood thresholds 0.0 to 0.9. G = cells grown with growth factor; N = cells grown without growth factor. **B.** Cell fractions of a grade 3 oligodendroglioma (BT237) ccAFv2 cell cycle classifications using likelihood thresholds 0.0 to 0.9. G = cells grown with growth factor; N = cells grown without growth factor.  

 

**Supplementary Figure S6****.** Cell cycle regression. **A.** U5-hNSC PCA plot colorized by ccAFv2. **B.** U5-hNSC with S and G2/M ccAFv2 classes regressed PCA plot colorized by ccAFv2. **C** U5-hNSC with all cycling ccAFv2 classed regressed PCA plot colorized by ccAFv2. **D**. U5-hNSC PCA plot colorized by ccSeurat. **E**. U5-hNSC with S and G2/M ccSeurat classes regressed PCA plot colorized by ccSeurat. **F**. Venn diagram of ccAFv2 cell cycle state marker genes and ccSeurat cell cycle state marker genes.

**Supplementary Figure S7****.** Application of ccAFv2 to quiescent (qNSC) and activated (aNSC) neural stem cells from Llorens‐Bobadilla et al, 2015. **A.** Cell fractions of qNSC1 ccAFv2 cell cycle classifications using likelihood thresholds 0.0 to 0.9. **B.** Cell fractions of qNSC2 ccAFv2 cell cycle classifications using likelihood thresholds 0.0 to 0.9. **C.** Cell fractions of aNSC1 ccAFv2 cell cycle classifications using likelihood thresholds 0.0 to 0.9. **D.** Cell fractions of aNSC2 ccAFv2 cell cycle classifications using likelihood thresholds 0.0 to 0.9.

**Supplementary Figure S8****.** Application of the R and python-based ccAFv2. **A.** Cell percentages of *in vivo* hNSCs from PCW8 from Zeng et al, 2023 of ccAFv2 cell cycle state classifications using the R package Seurat v4 and v5, and Python package Scanpy.  

Augustus M, Pineau D, Aimond F, Azar S, Lecca D, Scamps F, Muxel S, Darlix A, Ritchie W, Gozé C, et al. 2021. Identification of CRYAB+ KCNN3+ SOX9+ Astrocyte-Like and EGFR+ PDGFRA+ OLIG1+ Oligodendrocyte-Like Tumoral Cells in Diffuse IDH1-Mutant Gliomas and Implication of NOTCH1 Signalling in Their Genesis. *Cancers* **13**: 2107.

Cebrian-Silla A, Nascimento MA, Redmond SA, Mansky B, Wu D, Obernier K, Romero Rodriguez R, Gonzalez-Granero S, García-Verdugo JM, Lim DA, et al. 2021. Single-cell analysis of the ventricular-subventricular zone reveals signatures of dorsal and ventral adult neurogenesis eds. J.G. Gleeson, M.E. Bronner, and D. Van der Kooy. *eLife* **10**: e67436.

Dulken BW, Leeman DS, Boutet SC, Hebestreit K, Brunet A. 2017. Single-Cell Transcriptomic Analysis Defines Heterogeneity and Transcriptional Dynamics in the Adult Neural Stem Cell Lineage. *Cell Rep* **18**: 777–790.

Hafemeister C, Satija R. 2019. Normalization and variance stabilization of single-cell RNA-seq data using regularized negative binomial regression. *Genome Biol* **20**: 296.

Herculano-Houzel S, Lent R. 2005. Isotropic Fractionator: A Simple, Rapid Method for the Quantification of Total Cell and Neuron Numbers in the Brain. *J Neurosci* **25**: 2518–2521.

Llorens-Bobadilla E, Zhao S, Baser A, Saiz-Castro G, Zwadlo K, Martin-Villalba A. 2015. Single-Cell Transcriptomics Reveals a Population of Dormant Neural Stem Cells that Become Activated upon Brain Injury. *Cell Stem Cell* **17**: 329–340.

Nowakowski TJ, Bhaduri A, Pollen AA, Alvarado B, Mostajo-Radji MA, Di Lullo E, Haeussler M, Sandoval-Espinosa C, Liu SJ, Velmeshev D, et al. 2017. Spatiotemporal gene expression trajectories reveal developmental hierarchies of the human cortex. *Science* **358**: 1318–1323.

O’Connor SA, Feldman HM, Arora S, Hoellerbauer P, Toledo CM, Corrin P, Carter L, Kufeld M, Bolouri H, Basom R, et al. 2021. Neural G0: a quiescent-like state found in neuroepithelial-derived cells and glioma. *Mol Syst Biol* **17**: e9522.

Valério-Gomes B, Guimarães DM, Szczupak D, Lent R. 2018. The Absolute Number of Oligodendrocytes in the Adult Mouse Brain. *Front Neuroanat* **12**: 90.

Wolf FA, Angerer P, Theis FJ. 2018. SCANPY: large-scale single-cell gene expression data analysis. *Genome Biol* **19**: 15.

Zeng B, Liu Z, Lu Y, Zhong S, Qin S, Huang L, Zeng Y, Li Z, Dong H, Shi Y, et al. 2023. The single-cell and spatial transcriptional landscape of human gastrulation and early brain development. *Cell Stem Cell* **30**: 851-866.e7.

Zhang Q, Wu X, Fan Y, Jiang P, Zhao Y, Yang Y, Han S, Xu B, Chen B, Han J, et al. 2021. Single‐cell analysis reveals dynamic changes of neural cells in developing human spinal cord. *EMBO Rep* **22**: e52728.

Zhang Y, Sloan SA, Clarke LE, Caneda C, Plaza CA, Blumenthal PD, Vogel H, Steinberg GK, Edwards MSB, Li G, et al. 2016. Purification and characterization of progenitor and mature human astrocytes reveals transcriptional and functional differences with mouse. *Neuron* **89**: 37–53.
